# Supplementary material for: Widespread perturbation of ETS factor binding sites in cancer
Source: Nat Commun. 2023 Feb 17;14:913. doi: 10.1038/s41467-023-36535-8 (PMC9938127; doi:10.1038/s41467-023-36535-8)
Supplement: Supplementary file 1 — Supplementary information [file 41467_2023_36535_MOESM1_ESM.pdf]

Supplementary information

Widespread perturbation of ETS factor binding sites in cancer

#Carrasco Pro S[1], #Hook H[2], Bray D[1], Berenzy D[3], Moyer D[1], Yin M[2], Labadorf AT[4,5], Tewhey R[3], *Siggers T[1,2,6], *Fuxman Bass JI[1,2]

[1] Bioinformatics Program, Boston University, Boston, MA, USA.

[2] Department of Biology, Boston University, Boston, MA, USA.

[3] The Jackson Laboratory, Bar Harbor, ME, USA.

[4] Bioinformatics Hub, Boston University, Boston, MA, USA.

[5] Boston University School of Medicine, Department of Neurology, Boston, MA, USA.

[6] Biological Design Center, Boston University, Boston, MA, USA.

# these authors contributed equally

* these authors jointly supervised this work

Correspondence:

J.I.F.B.: fuxman@bu.edu

T.S.: tsiggers@bu.edu

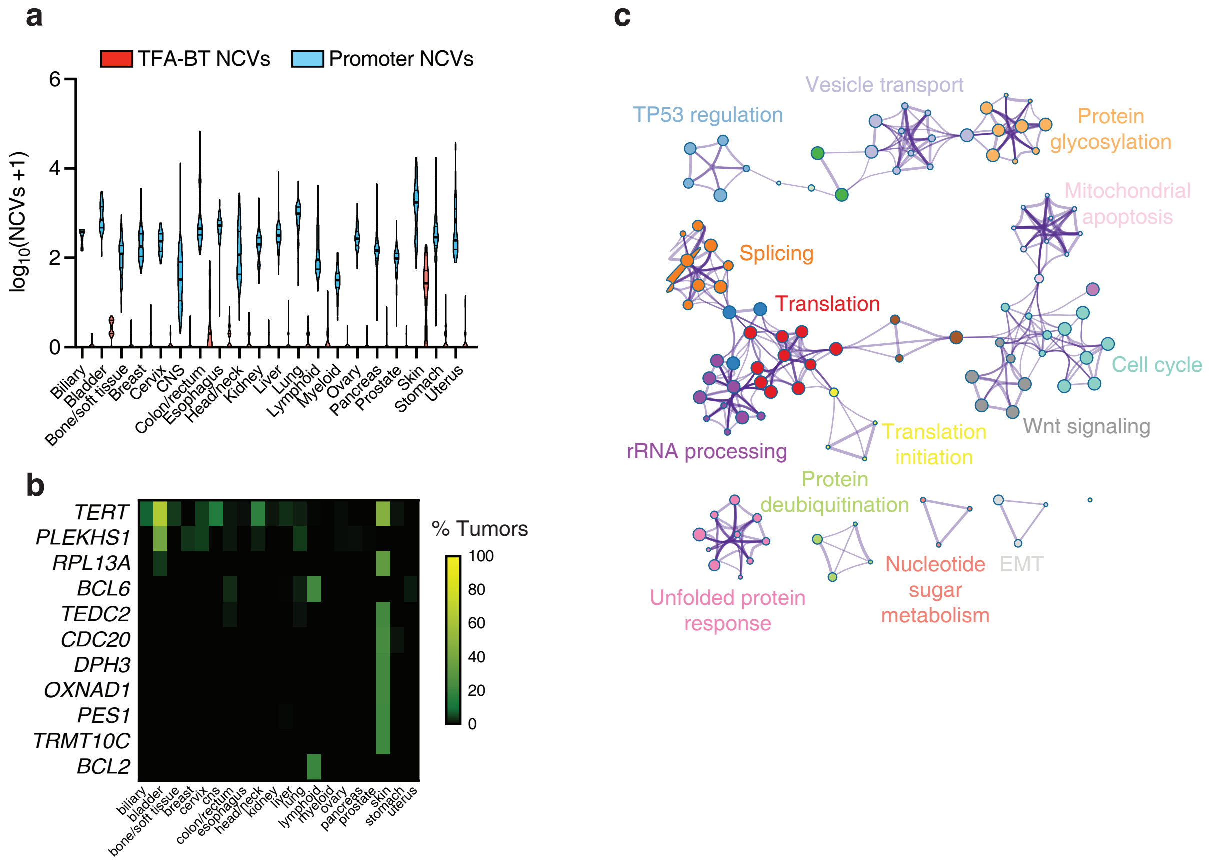

Supplementary Figure 1. Frequency of TFA-BT NCVs across cancers. (**a**) Number of TFA-BT NCVs and total promoter NCVs per patient for each cancer type: biliary (n = 12), bladder (n = 23), bone/soft tissue (n = 111), breast (n = 214), cervix (n = 20), CNS (n = 293), colon/rectum (n = 60), esophagus (n = 98), head/neck (n = 91), kidney (n = 189), liver (n = 339), lung (n = 86), lymphoid (n = 202), myeloid (n = 38), ovary (n = 113), pancreas (n = 323), prostate (n = 210), skin (n = 107), stomach (n = 74), uterus (n = 51). The violin plot contains lines indicating the first, second, and third quartiles. (**b**) Percentage of tumors per cancer type with TFA-BT mutations for each of the indicated genes. Only genes with TFA-BT NCVs in at least 5% of tumor samples in at least one cancer type are shown. (**c**) Metascape enrichment network showing gene ontologies significantly associated with TFA-BT genes. Each node represents a gene ontology term. Pairs of nodes with Kappa similarities aboved 0.3 are connected by edges and connected nodes share genes. Source data are provided as a Source Data file.

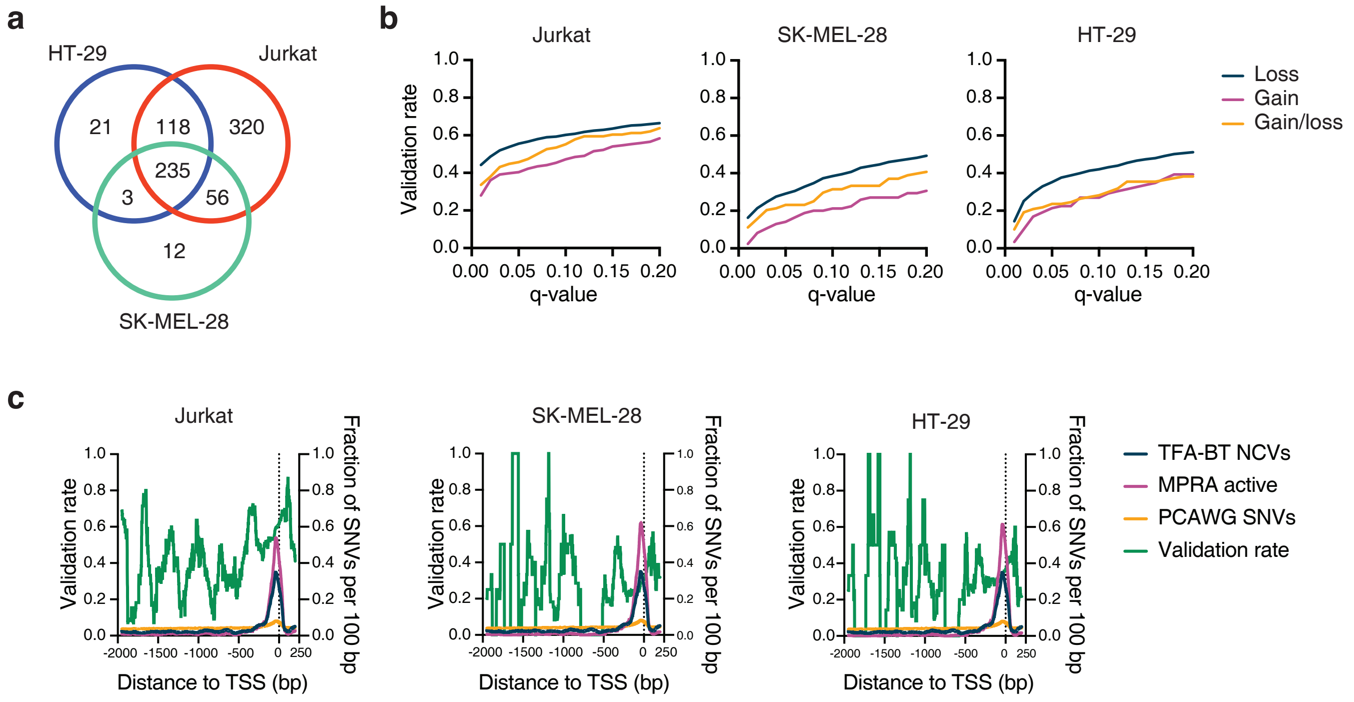

Supplementary Figure 2. TFA-BT NCVs validation rate. (**a**) Venn diagram showing the overlap between TFA-BT NCVs with significant allelic skew (q < 0.05) by MPRAs identified in Jurkat, HT-29, and SK-MEL-28 cells. (**b**) Fraction of TFA-BT NCVs associated with loss, gain, or gain and loss of TFBSs within MPRA active regions that show expression allelic skew at different q-value thresholds in Jurkat, SK-MEL-28, and HT-29 cells. (**c**) MPRA validation rate of TF-ABT NCVs based on the genomic distance to transcription sart site (TSS). The fraction of NCVs per 100 bp for TFA-BT NCVs, MPRA active TF-ABT NCVs, and SNVs in the PCAWG cohort are also indicated. Source data are provided as a Source Data file.

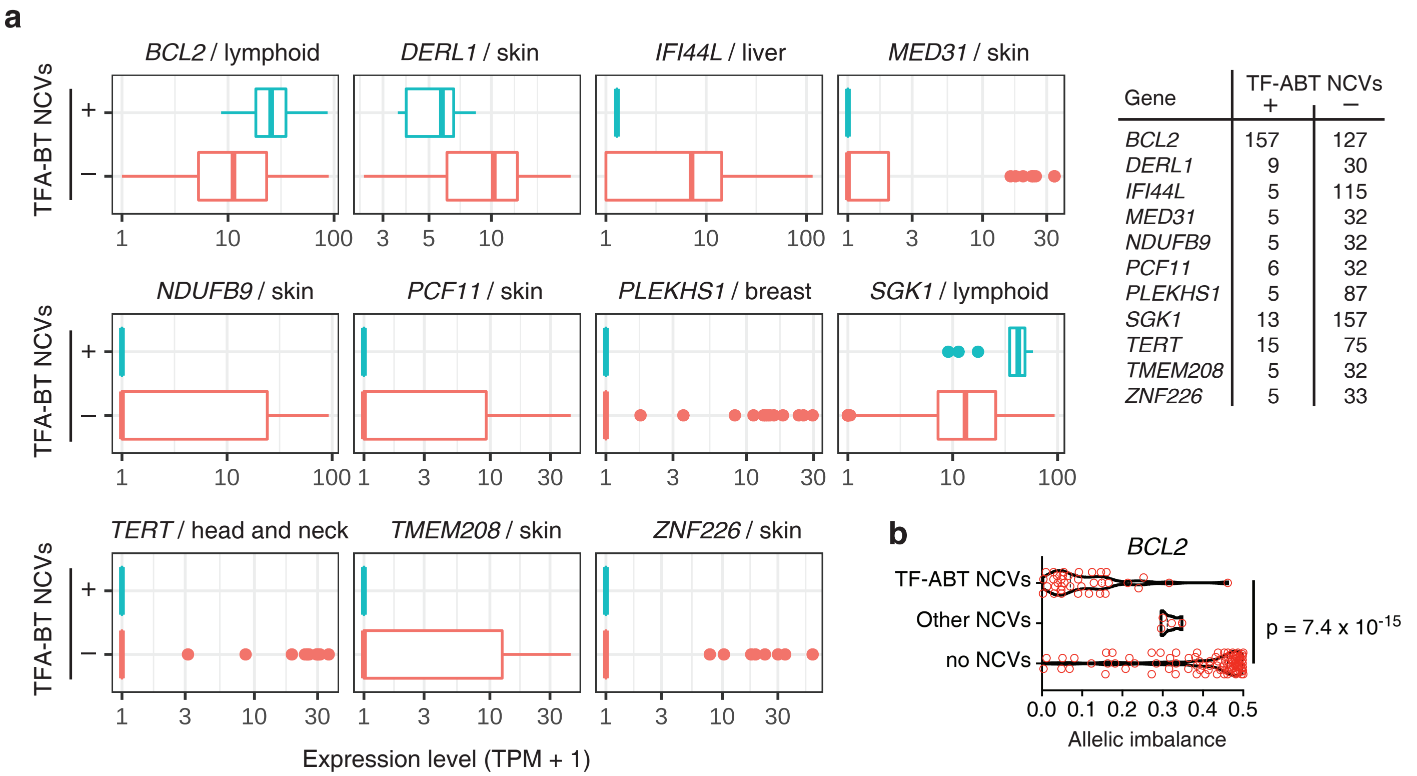

Gene	TF-ABT NCVs +	TF-ABT NCVs –
BCL2	157	127
DERL1	9	30
IFI44L	5	115
MED31	5	32
NDUFB9	5	32
PCF11	6	32
PLEKHS1	5	87
SGK1	13	157
TERT	15	75
TMEM208	5	32
ZNF226	5	33

Supplementary Figure 3. Effect of TFA-BT NCVs on gene expression. (**a**) Expression levels of TFA-BT genes in the corresponding cancer types for tumor samples with and without TFA-BT NCVs in the indicated gene. Each box spans from the first to the third quartile, the horizontal lines inside the boxes indicate the median value, the whiskers indicate 1.5x the interquartile range, individual points indicate data outside of the whiskers. Statistical significance determined by two-sided Mann-Whitney's U tests. Only gene-cancer type associations with an FDR < 0.05 and at least five tumor samples with TFA-BT NCVs are included in the figure. The table indicates the number of patient samples with (+) or without (–) TF-ABT NCVs in the corresponding gene promoters. (**b**) *BCL2* expression allelic imbalace for tumor samples with TFA-BT NCVs, other NCVs, or no NCVs in the *BCL2* promoter. Source data are provided as a Source Data file.

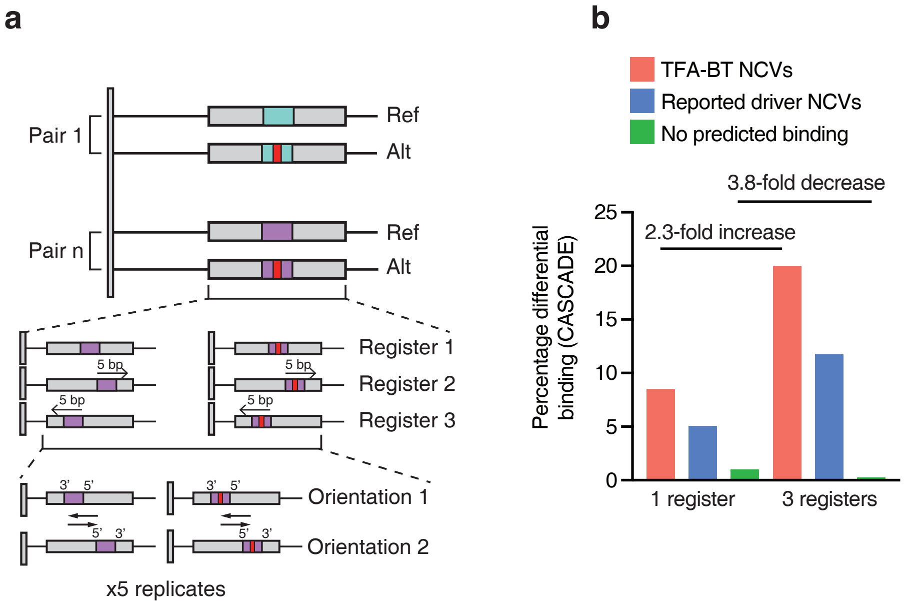

Supplementary Figure 4. CASCADE experiment design. (**a**) Outline of CASCADE experiments. Each reference (Ref) and alternative (Alt) NCV allele was tested in both orientations relative to the glass slide and using three registers: center, 5 bp shift upstream and 5 bp shift downstream. Each of these sequences was tested in five replicates placed in different positions in the array. (**b**) CASCADE performance improvement using three versus one register. The bar graph depicts the percentace of TFA-BT, reported driver, and no prodicted binding NCVs that show differential cofactor recruitment when tested in one or three registers.

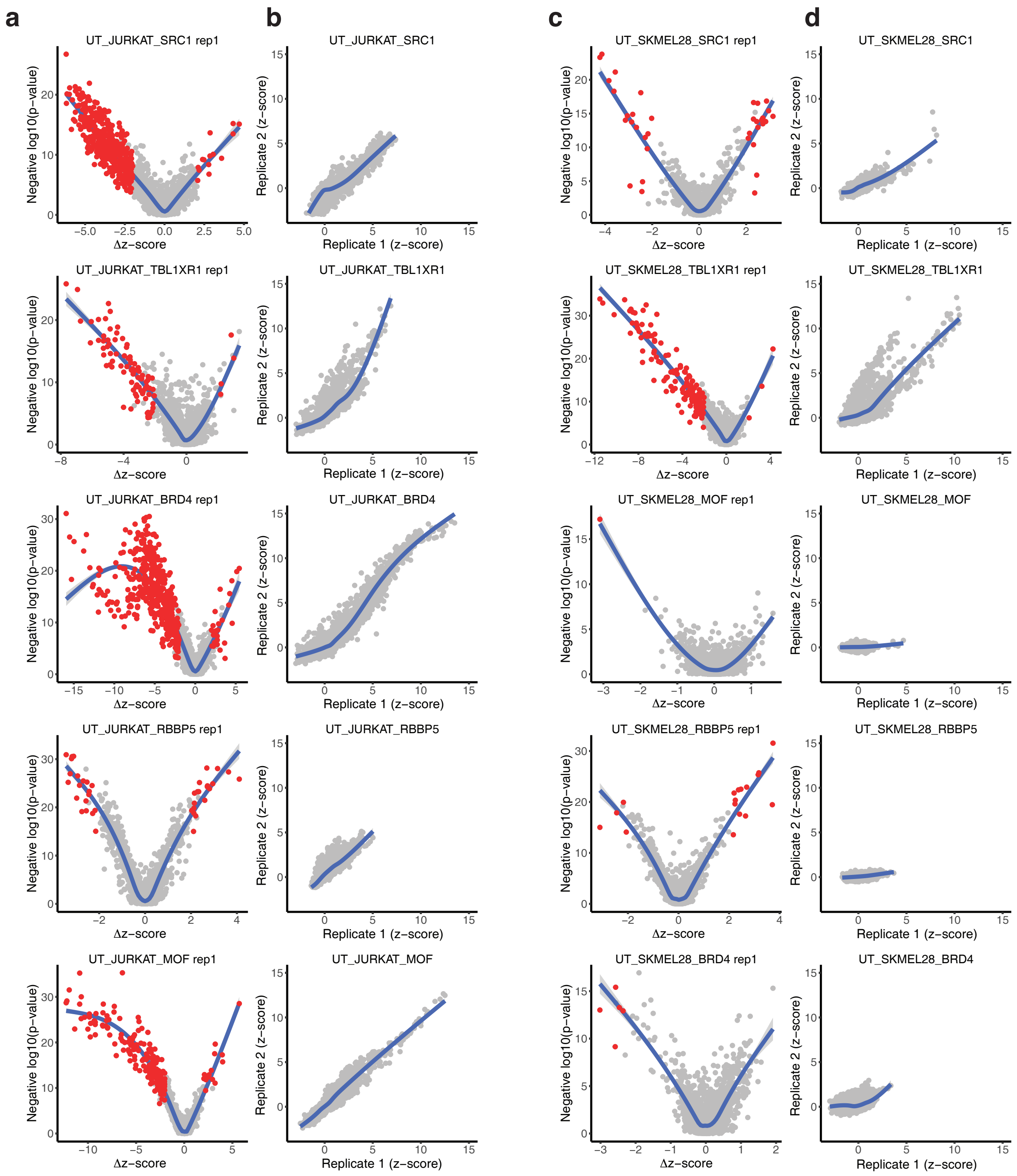

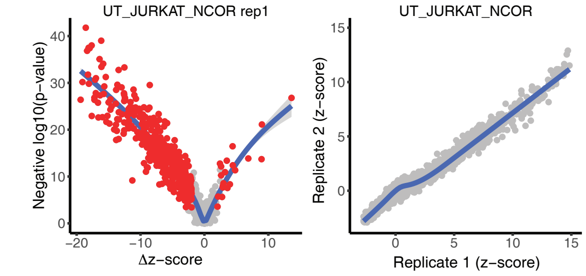

Supplementary Figure 5. Identification of TFA-BT NCVs that alter COF recruitment. (**a**, **c**) Impact of TFA-BT NCVs on COF recruitment using Jurkat (**a**) and SK-MEL-28 (**c**) nuclear extracts. Impact is quantified with -$\log_{10}$(p-value) of the COF recruitment to the different probe sets and the difference in PBM-determined Z-score between Ref and Alt alleles (Δz-score). The NCVs identified as significant are highlighted in red. (**b, d**) Reproducibity of CASCADE. Z-scores for each TFA-BT NCV are compared between biological replicates performed using Jurkat (**b**) and SK-MEL-28 (**d**) nuclear extracts. Source data are provided as a Source Data file.

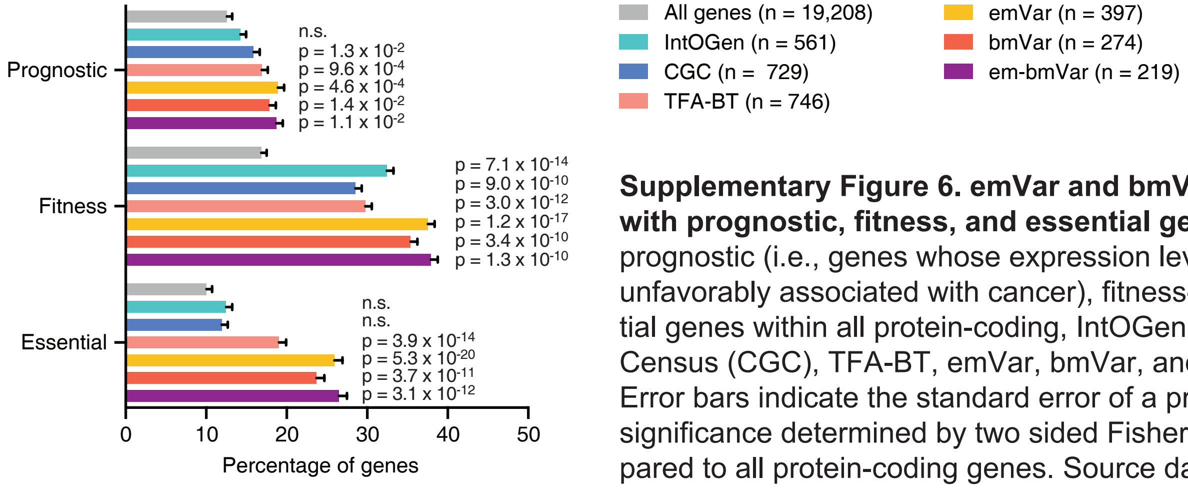

Supplementary Figure 6. emVar and bmVar gene association with prognostic, fitness, and essential genes. Percentage of prognostic (i.e., genes whose expression levels are favorably or unfavorably associated with cancer), fitness-related, and essential genes within all protein-coding, IntOGen, Cancer Gene Census (CGC), TFA-BT, emVar, bmVar, and em-bmVar genes. Error bars indicate the standard error of a proportion. Statistical significance determined by two sided Fisher's exact test compared to all protein-coding genes. Source data are provided as a Source Data file.

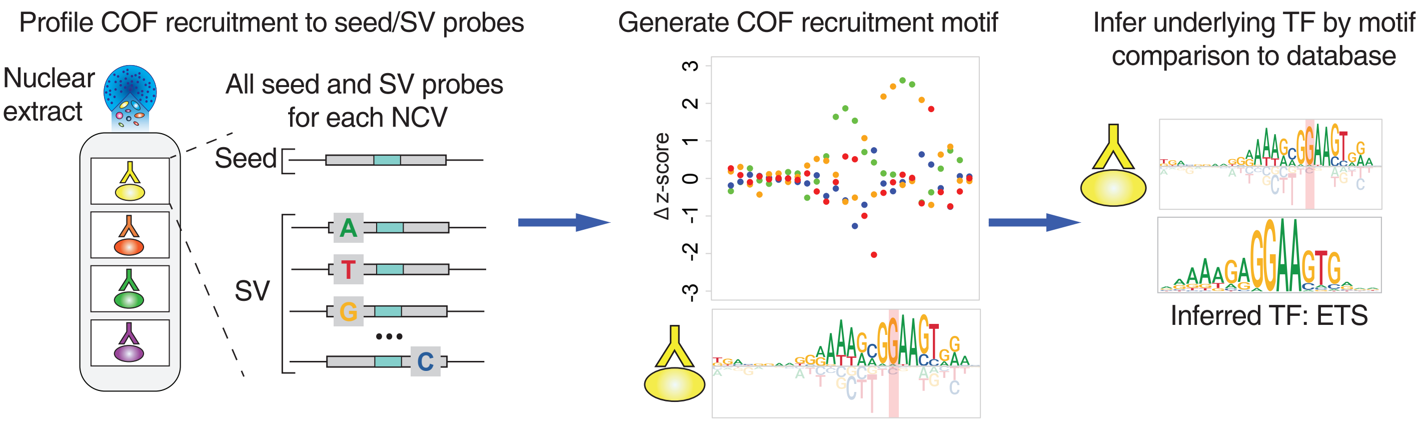

Supplementary Figure 7. Identification of underlying DNA motifs affecting COF recruitment. Outline illustrating the CASCADE approach to determine the DNA motifs affecting COF recruitment. COF recruitment is assayed to a “seed” probe containing the Ref or Alt NCV sequences in the genomic context and all single variant (SV) probes. The confetti plots show COF recruitment preferences to single variant probes along DNA sequence. Preferences are transformed to a COF recruitment motif. COF recruitment motifs are matched to TF motif databases to infer the identity of the TF recruiting the COF.

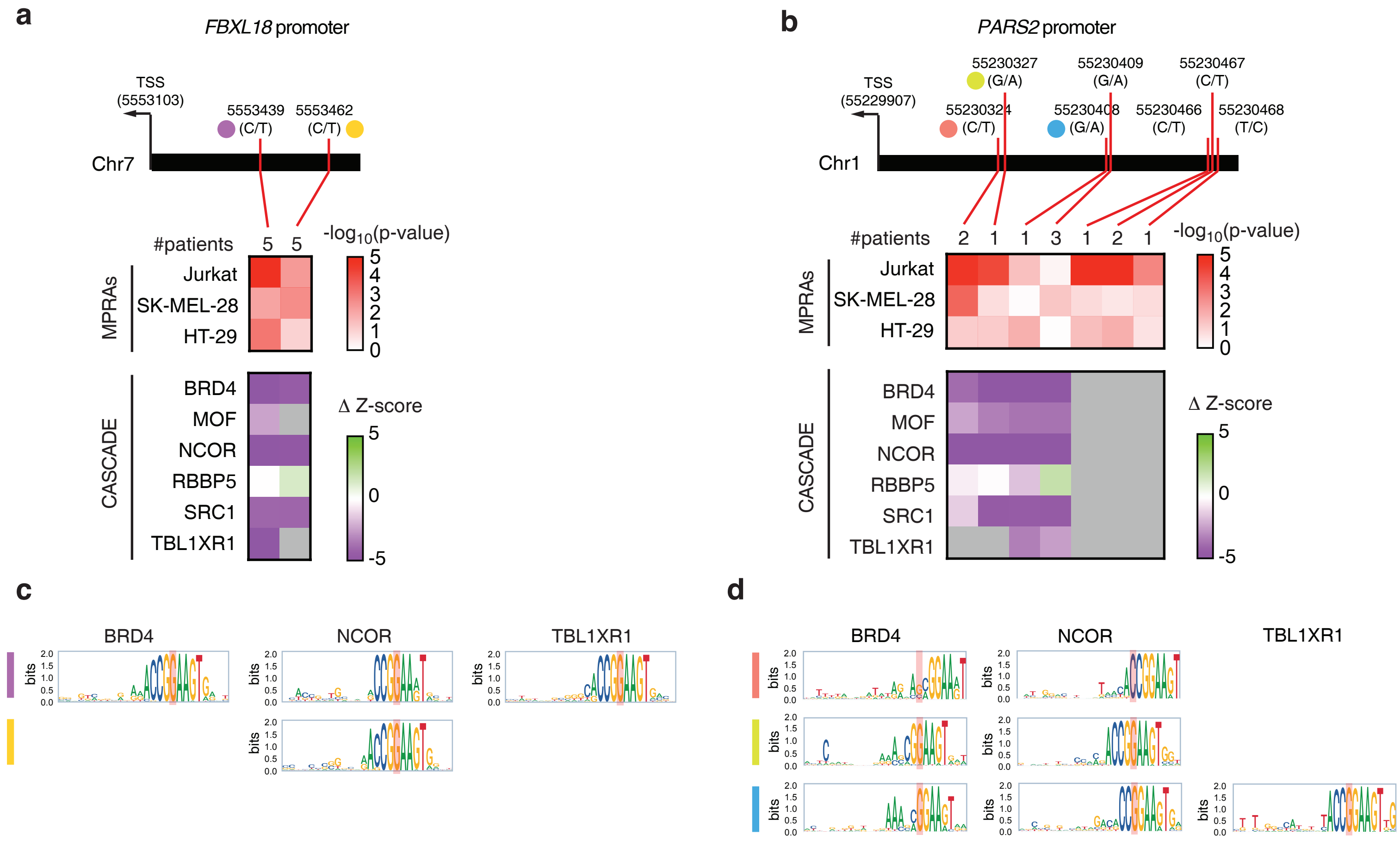

Supplementary Figure 8. Altered transcriptional activity and COF recruitment within promoters. (**a-b**) Changes in MPRA activity and COF recruitment for TF-ABT NCV in the (**a**) *FBXL18* and (**b**) *PARS2* promoters. The top heatmaps show the log10(p-value) of expression allelic skew in MPRA in Jurkat, SK-MEL-28, and HT-29 cells is indicated. P values were calculated using two-sided Student's t-test. The bottom heamaps show the altered COF recruitment by CASCADE, which is indicated as Δz-score. Gray cells indicate cases where the COF was not recruited to either NCV allele. Numbers at the top of the heatmaps indicate the number of patients in PCAWG carrying the indicated NCV. (**c-d**) COF recruitment motifs determined by single nucleotide variant scanning using CASCADE for the NCVs indicated in **a-b**. Source data are provided as a Source Data file.

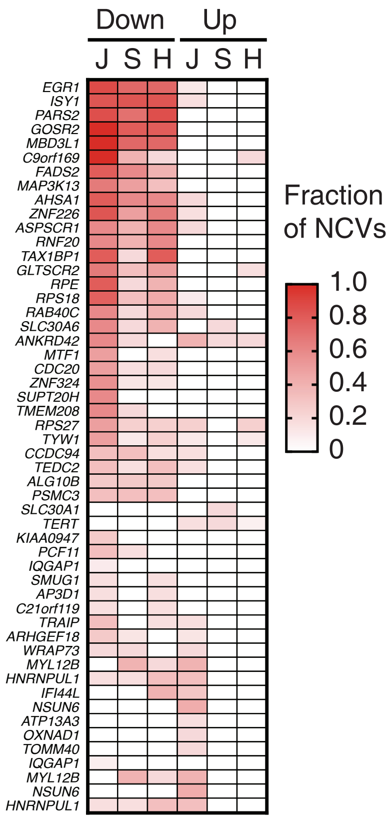

Supplementary Figure 9. TFA-BT NCVs associated with transcriptional activation and repression. Each cell in the heatmap represents the number of TFA-BT NCVs with increased (up) or decreased (down) transcriptional activity in MPRAs relative to the number of TFA-BT NCVs in MPRA active regions for the indicated gene and indicated cell line. J – Jurkat, S – SK-MEL-28, H – HT-29. Only genes with at least four TF-ABT NCVs in MPRA active regions are shown. Source data are provided as a Source Data file.

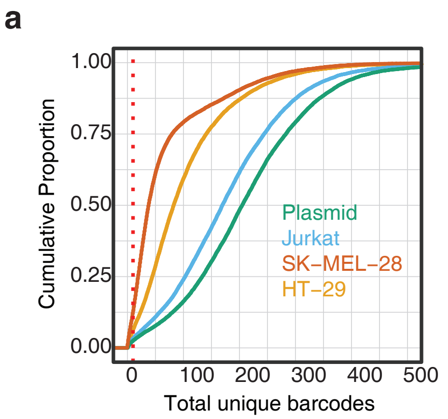

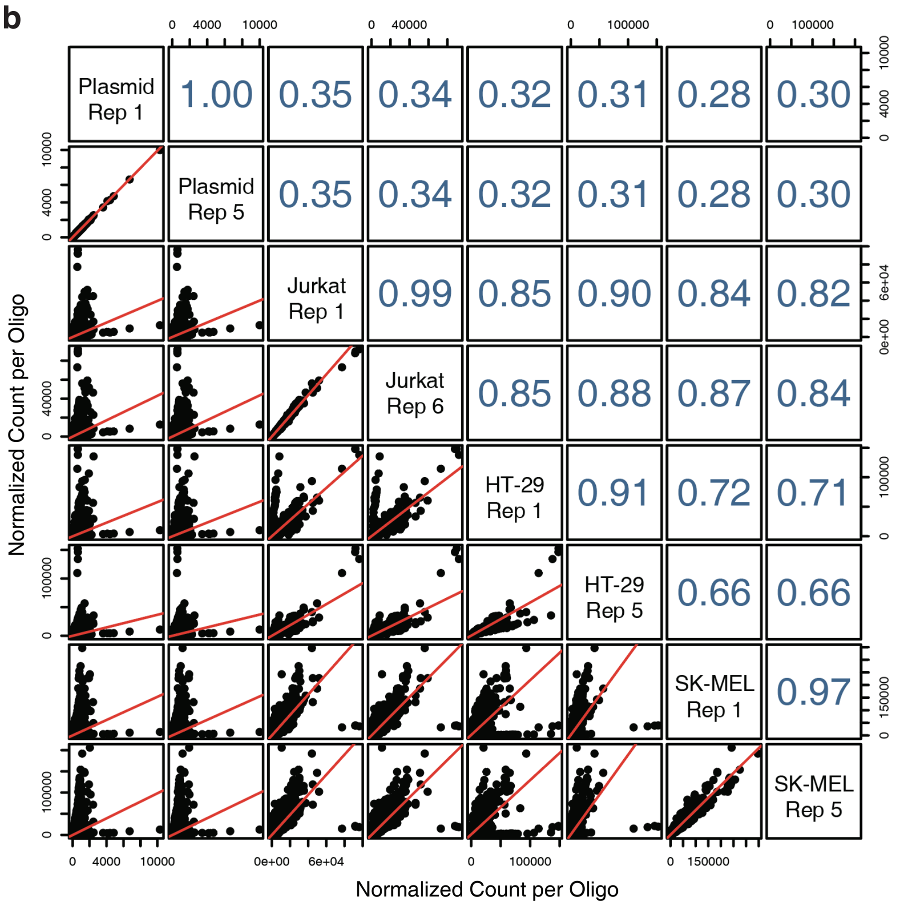

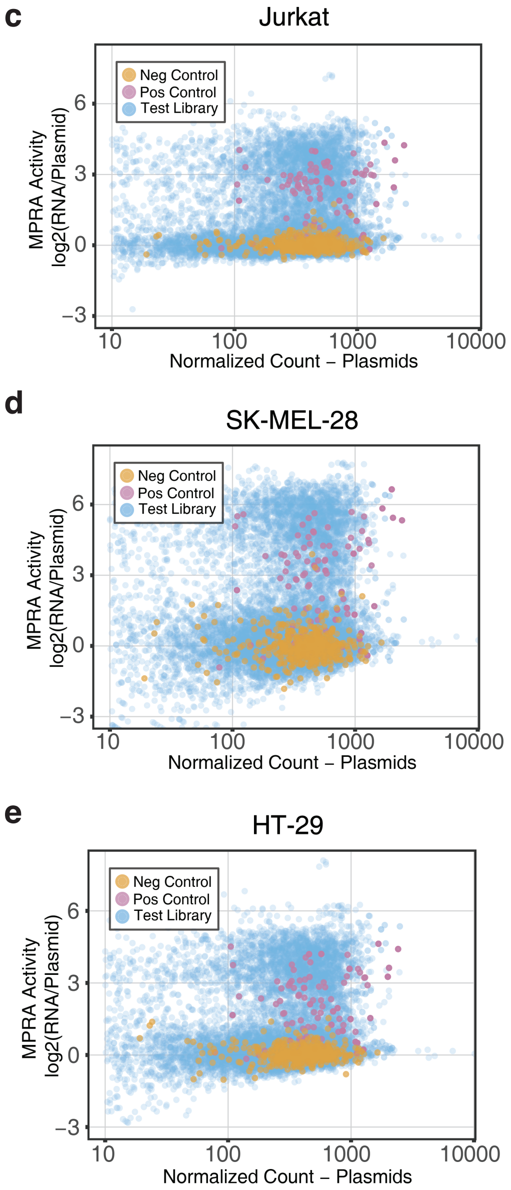

Supplementary Figure 10. MPRA quality control. (**a**) Cumulative distribution of MPRA oligos ranked by the number of unique barcodes (x-axis) detected across all replicates in the three cell types and plasmid library. Red dashed line marks 10 barcodes. (**b**) Correlation matrix of the summed barcode counts for each oligo. Two representative biological replicates (first and last) for each cell line and the plasmid library are plotted. Lower left matrix is a scatter plot of normalized oligo counts. A best fit linear regression is provided for each plot as a solid red line. Upper right matrices are pearson correlations (r) for each pairwise comparison. (**c-e**) Scatter plots for Jurkat (**c**), SK-MEL-28 (**d**), and HT-29 (**e**) with normalized oligo counts in the plasmid library on the x-axis and MPRA activity (Log2(RNA/Plasmid)) on the y-axis. Points are colored to denote specific project categories; test oligos (blue), positive activity controls (purple), and negative activity controls (orange).

A	
#120	CCTCGATGTTGTGGCGGGTCTTGAAGTTCACCTTG/3BioTEG/
#123	CCAGGATGTTGCCGTCCTCCTTGAAGTCGATGCCC/3BioTEG/
#136	CGCCGTAGGTGAAGGTGGTCACGAGGGTGGGCCAG/3BioTEG/
#781	ACTGGAGTTCAGACGTGTGCTCTTCCGATCTCGCCCTGAGCAAAGACC
#782	ACTCTTTCCCTACACGACGCTCTTCCGATCT
P7	CAAGCAGAAGACGGCATACGAGAT(NNNNNNNN)GTGACTGGAGTTCAGACGTGTGCTCTTCCGATCT
P5	AATGATACGGCGACCACCGAGATCTACAC(NNNNNNNN))ACACTCTTTCCCTACACGACGCTCTTCCGATC

B		
	P7 Index	P5 Index
Jurkats Rep 1	AGATGTGC	AACCTCTT
Jurkats Rep 2	TCAGCGAA	CGCATATT
Jurkats Rep 3	GAATTGCT	CTGCTCCT
Jurkats Rep 4	AGGATGTG	GCTGCACT
Jurkats Rep 5	CTAACTGG	CCTGTCAT
Jurkats Rep 6	ACATCCTT	CACTTCAT
HT-29 Rep 1	CTATTCAA	TCCATAAC
HT-29 Rep 2	GACCGAGA	CTGACATC
HT-29 Rep 3	CCTTGCTG	CCTCTAAC
HT-29 Rep 4	CTAGGTTC	CTGGTATT
HT-29 Rep 5	AGCTCTGG	CGAACTTC
Mel-28 Rep1	CCTGGTAG	CAACTGAT
Mel-28 Rep2	CAGTTGGT	GGCAATAC
Mel-28 Rep3	TACTTGCA	CCAACTAA
Mel-28 Rep4	TCGCACCT	CTTCTGGC
Mel-28 Rep5	ACATAGCG	TCCGCATA

Supplementary Table 1: Primers and sequencing indices used. (A) Primers used in MPRA experiments and (B) Illumina Adaptor/Index sequences.

Antibody	Vendor	Catalog Number
**Primary Antibodies**		
RBBP5	ThermoFisher	A300-109A
TBL1XR1	Santa Cruz Biotechnology	sc-100908
BRD4	ThermoFisher	A301-985A50
SRC1	Santa Cruz Biotechnology	sc32789x
NCOR	ThermoFisher	A301-145A
MOF	ThermoFisher	A300-992A
**Secondary Antibodies**		
Donkey anti-goat IgG (H+L) Cross-Adsorbed Secondary Antibody, Alexa Fluor 488	ThermoFisher	A11055
Goat anti-mouse IgG (H+L) Highly Cross-Adsorbed Secondary Antibody, Alexa Fluor 488	ThermoFisher	A11029
Goat anti-rabbit IgG (H+L) Highly Cross-Adsorbed Secondary Antibody, Alexa Fluor 488	ThermoFisher	A11034
Goat anti-mouse IgG (H+L) Highly Cross-Adsorbed Secondary Antibody, Alexa Fluor 647	ThermoFisher	A32728
Goat anti-rabbit IgG (H+L) Highly Cross-Adsorbed Secondary Antibody, Alexa Fluor 647	ThermoFisher	A32733

Supplementary Table 2: Antibodies used in the CASCADE experiments. List of antibodies, vendors, and catalog numbers used in the CASCADE experiments.
