# Supplementary material for: Widespread perturbation of ETS factor binding sites in cancer
Source: Nat Commun. 2023 Feb 17;14:913. doi: 10.1038/s41467-023-36535-8 (PMC9938127; doi:10.1038/s41467-023-36535-8)
Supplement: Supplementary file 3 — Description to Additional Supplementary Information [file 41467_2023_36535_MOESM3_ESM.pdf]

Description of Additional Supplementary Files

Supplementary Data 1: List of TF-ABT NCVs. Catalogue of TFA-BT NCVs, associated genes, TFs, and frequencies per cancer-type.

Supplementary Data 2: MPRA results. MPRA differential activity and statistical significance for TF-ABT and control NCVs in MPRA active regions.

Supplementary Data 3: CASCADE data for the differential binding screen of cofactors to Ref/Alt alleles. Z-scores for the Ref and Alt alleles are provided for sequences evaluated in both orientations and NCVs evaluated in three registers (-5, 0 and 5). Statistical significance between alleles as determined using Fisher's method combining six independent p-value determined using two-sided Student's t-test.

Supplementary Data 4: CASCADE-determined Motif Matrices for NCV loci. 26-bp long motif matrices are shown in 4-row format. Motif matrices are preceded by a header line containing an ID and a Microarray REPLICATE indicator (rep=1 or rep=2).

Supplementary Data 5: ChIP-seq data. List ChIP-seq experiments downloaded from the ENCODE Project that were analyzed for allelic imbalance.

Supplementary Data 6: List of gene promoters. List of gene promoter coordinates used for TFA-BT predictions. The 'Promoters' worksheet contains the coordinates of promoters and alternative promoters for protein-coding genes. The 'promoters - CDS' worksheet contains promoter fragments excluding regions corresponding to coding sequences.

Supplementary Data 7: MPRA sequences. Oligonucleotide sequences used in the MPRA experiments corresponding to TF-ABT and control NCVs.

Supplementary Data 8: List of Probe Sequences on Microarray Design #1 for determining motifs at NCV loci. The design includes 2,956 Ref/Alt paired probe sets: 2,555 TFA-BT NCVs, 17 literature-reported driver NCVs, and 384 background NCVs.

Supplementary Data 9: List of Probe Sequences on Microarray Design #2 for determining motifs at NCV loci. The design includes probes to evaluate motifs at 359 NCVs identified as significant by both CASCADE (differential COF recruitment using Design 1 microarray) and MPRAs (differential gene expression).
